# Health Outcome after Major Trauma: What Are We Measuring?

**DOI:** 10.1371/journal.pone.0103082

**Published:** 2014-07-22

**Authors:** Karen Hoffman, Elaine Cole, E. Diane Playford, Eva Grill, Helene L. Soberg, Karim Brohi

**Affiliations:** 1 Centre for Trauma Sciences, Blizard Institute of Cell and Molecular Science, Barts and the London School of Medicine and Dentistry, Queen Mary University of London, London, United Kingdom; 2 University College London (UCL) institute of Neurology, National Hospital for Neurology and Neurosurgery, Queen Square, London, United Kingdom; 3 Ludwig-Maximilians-Universität Munich, Institute for Medical Informatics, Biometry and Epidemiology (IBE), Munich, Germany; 4 Department of Physical Medicine and Rehabilitation, Oslo University Hospital, Oslo, Norway; Oregon Health & Science University, United States of America

## Abstract

**Importance:**

Trauma is a global disease and is among the leading causes of disability in the world. The importance of outcome beyond trauma survival has been recognised over the last decade. Despite this there is no internationally agreed approach for assessment of health outcome and rehabilitation of trauma patients.

**Objective:**

To systematically examine to what extent outcomes measures evaluate health outcomes in patients with major trauma.

**Data Sources:**

MEDLINE, EMBASE, and CINAHL (from 2006–2012) were searched for studies evaluating health outcome after traumatic injuries.

**Study selection and data extraction:**

Studies of adult patients with injuries involving at least two body areas or organ systems were included. Information on study design, outcome measures used, sample size and outcomes were extracted. The World Health Organisation International Classification of Function, Disability and Health (ICF) were used to evaluate to what extent outcome measures captured health impacts.

**Results:**

34 studies from 755 studies were included in the review. 38 outcome measures were identified. 21 outcome measures were used only once and only five were used in three or more studies. Only 6% of all possible health impacts were captured. Concepts related to activity and participation were the most represented but still only captured 12% of all possible concepts in this domain. Measures performed very poorly in capturing concepts related to body function (5%), functional activities (11%) and environmental factors (2%).

**Conclusion:**

Outcome measures used in major trauma capture only a small proportion of health impacts. There is no inclusive classification for measuring disability or health outcome following trauma. The ICF may provide a useful framework for the development of a comprehensive health outcome measure for trauma care.

## Background and Introduction

Trauma is a major contributor to the world's burden of disability, and responsible for the loss of more Disability-Adjusted Life Years than any other disorder [1]. Injury can result in long-standing adverse effects on patients' overall health and quality of life [2]. Understanding the full extent of the impact of trauma on an individual's health has the potential to direct treatment, rehabilitation and social care services [3]. Capturing health impact on a population basis is important for health services design and delivery, resource allocation and for future research and development [4]. The comprehensive measurement of function, disability, health and quality of life outcomes after injury is of fundamental importance to trauma care.

While there are tools to measure health outcomes [5] it is not clear to what extent they are able to capture the full range of effects injury may have on health and well-being. There are no valid, internationally applied trauma-specific tools that have been designed to evaluate long term recovery [6], [7]. Generic measures have been used to assess rehabilitation and functional outcome after trauma [8]–[11], but there are no studies that examine how well these capture the range of health impacts that trauma patients may experience. The result of this is that there is no international consensus on which rehabilitation framework should be used in the evaluation of health outcomes after trauma [2], [12].

The International Classification of Function, Disability and Health (ICF) [13] is an internationally recognised framework that describes health and health related states and was developed in conjunction with the World Health Organisation. The ICF captures approximately 1400 health concepts and is recognised to encompass the breadth of potential health impacts of disease. The primary aim of this study was to assess the ability of measures currently used in major trauma outcome studies to capture the full range of patient important health impacts, using the ICF as a framework. We performed a systematic review of outcomes measures used in studies of function and disability after major trauma. We specifically evaluated to what degree and frequency three main health categories were evaluated - body functions, activities and participation and environmental facilitators or barriers.

## Methods

### Data sources and search strategy

**Figure 1:**
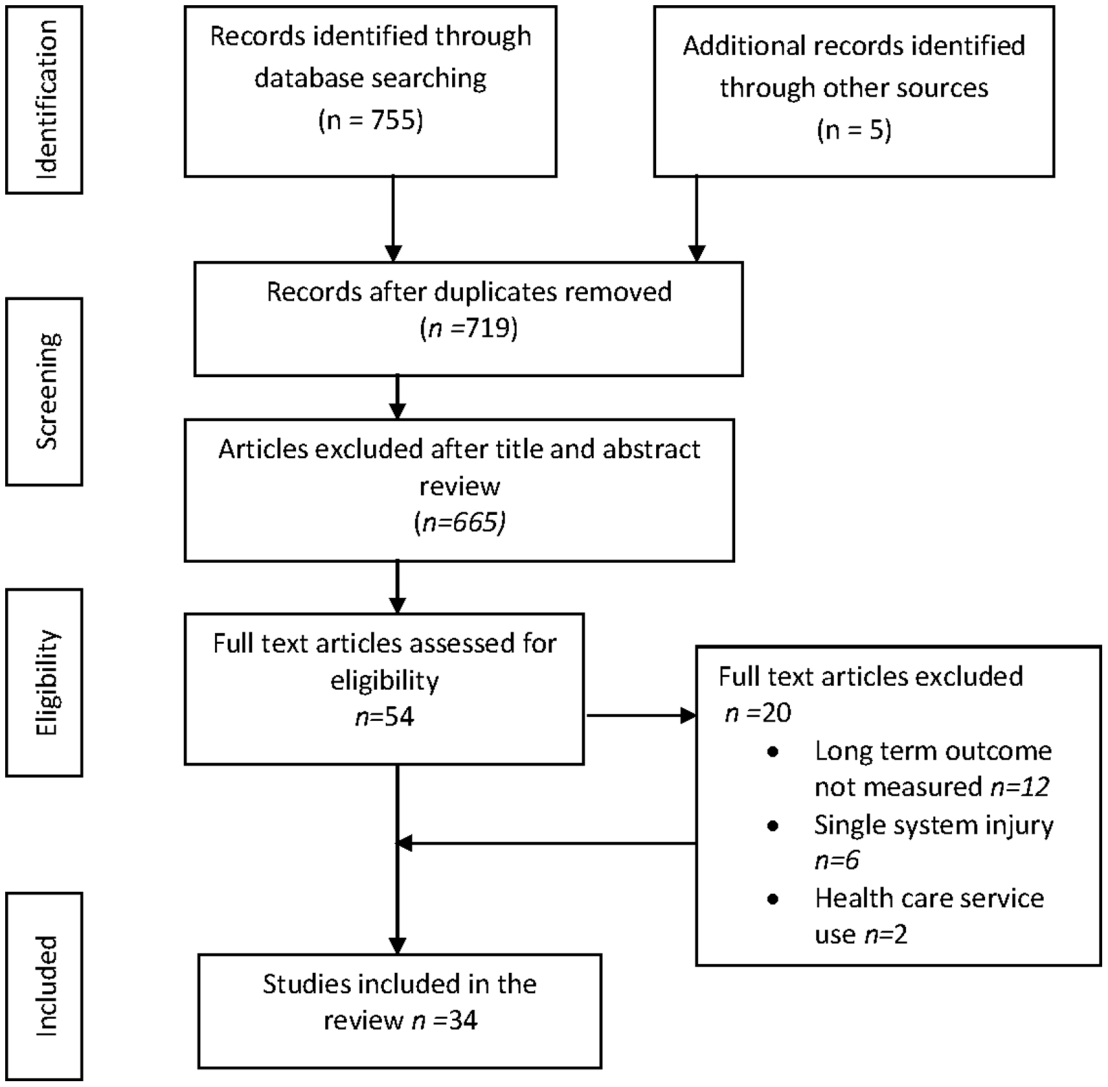
*PRISMA flow diagram of the screened and included papers. * Preferred Reporting Items for Systematic Reviews and Meta-Analyses.

Many trauma outcome studies prior to 2001 used outcome measures based on the International Classification of Impairment, Disability and Handicap framework (ICIDH-2) [14] rather than the ICF [13] which measures health. Published studies between and including 2006 and 2012 were included in the search in an attempt to capture outcome measures based on the new ICF classification. A 16-step electronic search strategy of English language studies was developed for Medline and adapted for EMBASE and Cumulative Index of Nursing and Allied Health Literature (CINAHL) databases. A combination of multiple search terms with four themes was used: major trauma (wound and injuries), outcome (outcome measures, tools, measures) quality of life and rehabilitation (Table S1). The PRISMA[15] process was used to identify suitable studies (Figure 1).

### Study selection and inclusion/exclusion criteria

The inclusion criteria were studies written in English, published in peer review journals that evaluated health or rehabilitation outcome following major traumatic injuries. We included randomised control trials, cross sectional and cohort studies of adult patients (≥18 years) with injuries involving at least two body areas or organ systems in order to exclude single system injuries. Excluded studies were those based on isolated spinal cord injuries or traumatic brain injuries as these would have different scopes and outcomes; case studies with less than 10 patients and studies that did not measure health outcome after major trauma. The study selection process is summarized in Figure 1.

### Screening and data extraction

All study titles and abstracts, including reference lists were screened by two independent researchers Karen Hoffman (KH) and Elaine Cole (EC). Once duplicates were removed, inclusion criteria were applied and studies for full text review were identified. Full text articles were reviewed by one reviewer (KH) and a random sample of 50% was also screened by the second reviewer (EC). Any discrepancies were resolved by both authors re-reviewing the study. In a second step we extracted outcome measures that occurred in at least three or more studies or those which are valid, trauma specific outcome measures, rather than generic health measures. The information extracted from studies included: Country of publication, study design, sample size and outcome measures used (Table 1, Table S3 and Table 2).

**Table 1:** Characteristics of studies included in the review.

Source	Study design	Number of participants	Country	Standardised outcome instruments
Ballabeni et al, 2011 [53]	Prospective cohort	391	Switzerland	Karasek's 31-item Job Content Questionnaire (JCQ)
Baranyi et al, 2010 [45]	Prospective cohort	52	Germany	German version of the Clinician-Administered PTSD Scale (CAPS)
				Syndrom-Kurz Test (SKT)
				Beck Depression Inventory (BDI),
				Impact of Events Sale (IES)
				Dissociative Experience Scale (DES)
				Medical Outcomes Study Short Form Health Survey (SF-36)
Christensen et al, 2011 [26]	RCT	347	Denmark	Polytrauma Outcome Chart consisting of the -
				Glasgow Outcome Scale (GOC)
				European Quality of Life Questionnaire (EQ-5D)
				SF-36
				Trauma Outcome Profile (TOP)
Derrett et al, 2010 [40]	Prospective cohort	111	New Zealand	World Health Organisation Disability Assessment Schedule II (WHODAS II)
				EQ-5D
Franzén et al, 2009 [28]	RCT	568	Sweden	EQ-5D
Gabbe et al, 2013 [39]	Prospective cohort	617	Australia	Study Short Form 12 (SF-12)
				Glasgow Outcome Scale-Extended (GOS-E)
Gabbe et al, 2012 [38]	Database review	4986	Australia	GOS-E
Gabbe et al,2006 [37]	Prospective cohort	662	Australia	Modified Functional Independence Measure (FIM)
Harris et al, 2008 [36]	Prospective cohort	355	Australia	SF-36
Holtslag et al, 2007 [47]	Prospective cohort	335	The Netherlands	Glasgow Outcome Scale (GOS)
				EuroQol (EQ-5D)
				Head injury symptom checklist (HISC)
Holtslag et al, 2006 [46]	Prospective cohort	186	The Netherlands	Glasgow Outcome Scale (GOS)
				Groningen Activity Restriction Score (GARS)
				Sickness Impact Profile-136 (SIP)
				SF-36
Jackson et al, 2007 [30]	Prospective cohort	58	USA	Informant Questionnaire of Cognitive Decline in the Elderly-Short Form (IQCODE-SF)
				SF-36
				Beck's Depression Inventory (BDI)
				Katz Index of Independence in Activities of Daily Living
				Davidson Trauma Scale (DTS)
				Beck's Anxiety Inventory
				Functional Activities Questionnaire ((FAQ)
				Awareness questionnaire
Kiely et al, 2006 [31]	Prospective cohort	123	USA	SF-36
				FIM
				Post-Traumatic Stress Disorder Checklist (PCL)
				Centre for Epidemiologic Studies Depression Scale (CES-D-10)
Langley et al, 2011 [41]	Prospective cohort	2856	New Zealand	EQ-5D
Livingston et al, 2009 [32]	Prospective cohort	100	USA	GOS
				FIM
				Modified FIM
Mackenzie et al, 2008 [33]	Retrospective cohort	1389	USA	SF-36
				Musculoskeletal Function Assessment (MFA) –mobility subscale
				Centre for Epidémiologique Studies Depression Scale (CESD-R)
Orwelius et al, 2012 [52]	Prospective cohort	108	Sweden	SF-36
Pape et al, 2010 [44]	Prospective cohort	637	Germany	SF-12
				Hannover Score for Poly-trauma Outcome (HASPOC)
Pirente et al, 2007 [27]	RCT	171	Germany	Beck's Depression Inventory (BDI)
				SF-36
				State-Trait Anxiety Inventory (STAI)
				Symptom Checklist 90-Revised (SCL 90R)
				Social support Questionnaire (Fragebogen zur Sozialen Unterstützung; F-SOZU-22)
Polinder et al, 2007 [48]	Prospective cohort	3231	The Netherlands	EQ-5D
Probst et al, 2010 [42]	Prospective cohort	637	Germany	Hannover Score for Poly-trauma Outcome
				Short form-12, HADS
Ringburg et al, 2011 [49]	Prospective cohort	246	The Netherlands	Health Utilities Index (HUI)
				EQ5D
Sayer et al, 2008 [34]	Retrospective cohort	188	USA	Functional Independence Measure (FIM)
Schwartz et al, 2007 [54]	Retrospective cohort	72	Israel	Functional Independence Measure (FIM)
				Impact of Events Scale (IES)
Siddharthan et al, 2008 [35]	Retrospective cohort	116	USA	FIM
Soberg et al, 2007 [50]	Prospective cohort	100	Norway	Brief Approach/Avoidance Coping Questionnaire
				Multidimensional Health Locus of Control
				Short Form-36
				WHODAS-ll
Soberg et al, 2007 [8]	Prospective cohort	105	Norway	Short Form (SF)-36
				WHODAS II
Soberg et al, 2010 [51]	Prospective cohort	99	Norway	SF-36
				Post-Traumatic Symptom Scale 10 (PTSS-10)
Soberg et al, 2012 [9]	Prospective cohort	105	Norway	SF-36
				WHODAS II
Steel et al, 2010 [29]	Prospective cohort	620	USA	SF-12
Sutherland et al, 2006 [24]	Prospective cohort	200	UK	General Health Questionnaire (GHQ)
				Sickness Impact Profile (SIP)
				Musculoskeletal Function Assessment (MFA)
				SF-36
Sutherland et al, 2011 [25]	Prospective cohort	104	UK	General Health Questionnaire (GHQ)
				Sickness Impact Profile (SIP)
				Musculoskeletal Function Assessment (MFA)
				SF-36
Van Aswegen et al, 2011 [55]	Prospective cohort	42	South Africa	SF-36
Zeckey et al, 2011 [43]	Prospective cohort	620	Germany	HASPOC
				SF-12
				Glasgow Outcome Scale (GOS)

**Table 2:** Overview of the thirty eight outcome measures identified in 34 studies.

Description of instruments	n	% of 34 studies
Medical Outcome Study Short Form Health Survey (SF-36)	14	41
European Quality of Life Questionnaire (EQ-5D)	7	21
Functional Independence Measure (FIM)	5	15
Glasgow Outcome Scale (GOS)	5	15
World Health Organisation Disability Assessment Schedule II (WHODAS II)	4	12
Hannover Score for Polytrauma Outcome (HASPOC)	3	9
Musculoskeletal Function Assessment (MFA)	3	9
Study Short Form 12 (SF-12)	3	9
Sickness Impact Profile (SIP)	3	9
Glasgow Outcome Scale-Extended (GOS-E)	2	6
Modified Functional Independence Measure (FIM)	2	6
Impact of Events Scale-Revised (IES-R)	2	6
Social Support Questionnaire (SSQ)	2	6
Beck's Depression Inventory (BDI)	2	6
General Health Questionnaire (GHQ)	2	6
Centre for Epidemiologic Studies Depression Scale (CES-D)	2	6
Awareness questionnaire	1	3
Beck's Anxiety Inventory	1	3
Brief Approach/Avoidance Coping Questionnaire	1	3
Davidson Trauma Scale (DTS)	1	3
Dissociative Experience Scale (DES)	1	3
Functional Activities Questionnaire (FAQ)	1	3
German version of the Clinician-Administered PTSD Scale (CAPS)	1	3
Groningen Activity Restriction Score (GARS)	1	3
Health Utilities Index (HUI)	1	3
Hospital Anxiety and Depression Scale (HADS)	1	3
Informant Questionnaire of Cognitive Decline in the Elderly-Short Form (IQCODE-SF)	1	3
Karasek's 31-item Job Content Questionnaire (JCQ)	1	3
Katz Index of Independence in Activities of Daily Living	1	3
Multidimensional Health Locus of Control	1	3
Post Traumatic Symptom Scale (PTSS-10)	1	3
Post-Traumatic Stress Disorder Checklist (PCL)	1	3
Social support Questionnaire (Fragebogen zur Sozialen Unterstützung; F-SOZU-22)	1	3
State-Trait Anxiety Inventory (STAI)	1	3
Symptom Checklist 90-Revised (SCL 90R)	1	3
Syndrom-Kurtz Test (SKT)	1	3
Trauma Outcomes Profile (TOP)	1	3

### ICF content analysis

The ICF classification is hierarchically organized with increasing levels indicating increasing degree of detail. It consist of four components, each denoted by an alphanumerical code starting with a lower case letter indicating the component, i.e., b for Body Functions, s for Body Structures, d for Activities and Participation and e for Environmental Factors. Each component consists of several chapters and each chapter has several levels of categories (second, third and fourth). Third and fourth levels are sub-categories of the overall second level category; for example, b1 Mental functions' (first/chapter level), b114 Orientation functions' (second level), b1142 Orientation to person (third level), b11420 Orientation to self (fourth level).

In a first step we identified which ICF categories and ICF chapters are represented within frequently used measures. The content of each outcome measure was linked to the ICF using established linking rules to identify how many ICF concepts are contained in each outcome measure [16], [17]. Each outcome measure had a different number of items or questions. For example, the Medical Outcome Study Short Form Health Survey (SF-36) [18] has 36 items (questions) compared to the Functional Independence Measure (FIM) [19] which has 18 items (questions). Each item was linked to one or more ICF categories depending on the number of meaningful concepts contained in the item. One item can contain one or more meaningful concepts. For example, ‘Do you get tired when walking’? ‘Get tired’ is a concept related to endurance and is linked to a category in the Body Function component [exercise tolerance: b455]. ‘Walking’ is a concept related to mobility and is linked to a category in the Activity and Participation component [walking: d450]. If an item in an instrument was too general it was added to the most suitable subheading of the ICF.

All identified measures were linked to the ICF by one reviewer (KH) and a random selection of forty percent of concepts was also linked and compared by a second reviewer (EC). Further validation was gained for two measures through comparison of reviewers linking results to published results in scientific publications [20]–[22]. Where items were linked to the third- and fourth-level categories they were aggregated to second level categories (Table S5). We also analysed to what degree measures coved concepts contained in the ICF. Content density, bandwidth and content diversity of measures were calculated to establish the breadth, depth and diversity of outcome measures in relation to the ICF [20]. Content density evaluates the ratio of the number of ICF categories contained per instrument in relation to the number of items in the instrument. Measures with smaller content density have fewer and less complex items, which makes these easier to use in clinical settings [23]. A content density of one indicates that each item in the measures represents one ICF category. Greater than one indicates that each item measures more than one ICF category. Content diversity measures the depth or detail of the instrument. A lower content diversity index indicates that several items and their concepts are dedicated to measure the same topic or ICF category [23]. Bandwidth (%) measures the breadth of the instrument. It calculates the percentage of ICF categories in each instrument in relation to the total number of ICF categories (1454 categories). As we focused on second level categories, we calculated bandwidth using 363, the total number of second level categories rather than all 1454 ICF categories. A larger bandwidth (%) indicates that a greater number of ICF categories are included in the instrument, thus greater ICF coverage. Results are summarised in Table 3.

**Table 3:** Content analysis of measures and individual ICF representation.

	SF-36	EQ-5D	FIM	GOS	WHODAS ll	TOP
Number of studies cited in	14	7	5	5	4	1
Number of items in measure	36	5	18	5	36	32
Total concepts per measure	53	10	37	9	34	107
ICF categories	23	10	29	9	24	47
						
Body Function	6	2	10	2	3	14
Body Structure	0	0	0	1	0	14
Activity and Participation	17	8	19	6	21	29
Environmental factors	0	0	0	0	0	4
						
Content density*	0.64	2.0	1.61	1.8	0.67	1.91
Bandwidth (%) **	6.3	2.8	8.0	2.5	6.6	16.8
Content diversity †	0.43	1.0	0.78	1.0	0.71	0.57

Content analysis of individual measures indicate a small ICF representation (bandwidth %). Environmental factors are only represented in one measure (TOP). The concepts contained in the TOP was also linked to the most ICF categories (n = 93) and the GOS had the least ICF categories.

*Content density – number of ICF categories/number items in an instrument.

** Bandwidth (%) - number of distinct ICF categories/total number of second level ICF categories (363) x100.

† Content diversity - total number of different ICF categories/number of meaningful concepts in the instrument.

In a second step we examined the most frequently represented ICF categories. This is calculated by relative frequencies of all ICF categories identified in measures to determine how often an ICF category is captured overall in trauma research. The observed number of categories was compared to the total number of categories for all measures included. For example, it is expected that the category *pain* (b280) is included in all measures. However, it may only occur in three of the six measures, thus a relative frequency of 50% (Table S4). This is useful to distinguish which ICF health concepts are captured by measures frequently used in trauma studies. An arbitrary cut off of 30% was chosen to classify the most frequently identified ICF categories (relative frequency) in each ICF component which are presented under chapter headings (Table S4).

## Results

The search identified 755 published articles. After duplicates were removed, 665 articles were excluded based on abstract reviews. A full text review of 54 articles led to the final inclusion of 34 articles (Figure 1). Excluded studies consisted of twelve that did not measure health outcome, six evaluated outcome of a single system rather than multiple injuries and two studies evaluated health care resource use rather than outcome. Two research groups reported results on the same cohort of patients at different times from injury in four studies [8], [9], [24], [25]. The cohort size decreased over time due to loss to follow up. Data on all four of these studies were included.

### Study characteristics

The overall qualitative characteristics of the evidence are presented in supporting information Table S2. Three of the 34 studies were RCT's, involving 1086 participants [26]–[28]. One described a specific rehabilitation intervention using cognitive behavioural therapy to reduce post-traumatic stress and improve quality of life [27]; the other study, evaluated the impact of recombinant Factor VII on long term health outcome after severe trauma [26]. The largest RCT (n = 568) evaluated the cost effectiveness of nurse led telephone follow-up to improve quality of life for discharged trauma patients. There were 26 prospective cohort studies involving a total of 12664 participants. The remainder were retrospective studies (n = 6751). Collectively the studies were conducted in different countries and continents: Seven in the USA [29]–[35]; six in Australasia (four in Australia and two in New Zealand) [36]–[41]; 19 in Europe (five in Germany [27], [42]–[45], four each in The Netherlands [46]–[49] and Norway [8], [9], [50], [51], two each in the UK [24], [25] and Sweden [28], [52], one each in Switzerland [53], Denmark [26] and one each in Israel [54] and South Africa [55] (Table 1, Table S3).

### Description of outcome measures

Thirty eight outcome measures were identified in 34 studies (Table 2). Twenty one outcome measures were used only once. Five outcome measures were used in three or more studies and two trauma specific tools were used in less than three studies. The most frequently used outcome measure was the Medical Outcome Study Short Form Health Survey (SF-36) [18] used in 14 studies. The other four generic outcome measurements were the European Quality of Life Questionnaire (EQ-5D)[56], used in seven studies; the Functional Independence Measure (FIM) [19] and the Glasgow Outcome Scale (GOS) [57] both used in 5 studies; and the World Health Organisation Disability Assessment Schedule (WHODAS ll) [58] used in four studies (Table 1 and 2). There were only two trauma-specific tools. The Hannover Score for Polytrauma Outcome (HASPOC) [59], was used in 3 studies and the Trauma Outcomes Profile (TOP)[60] in one study. The HASPOC was only used in Germany. We were unable to find an example of the HASPOC or information on the psychometric properties of this instrument. Therefore this instrument was excluded from ICF linking. Literature pertaining to the development and psychometric properties of the TOP are limited although a recent study concluded that the TOP has potential use in trauma populations but requires further validation [60]. Five generic and one trauma specific outcome measures were included in the analysis based on the inclusion criteria.

### Degree to which measures cover concepts contained in the ICF

**Figure 2:**
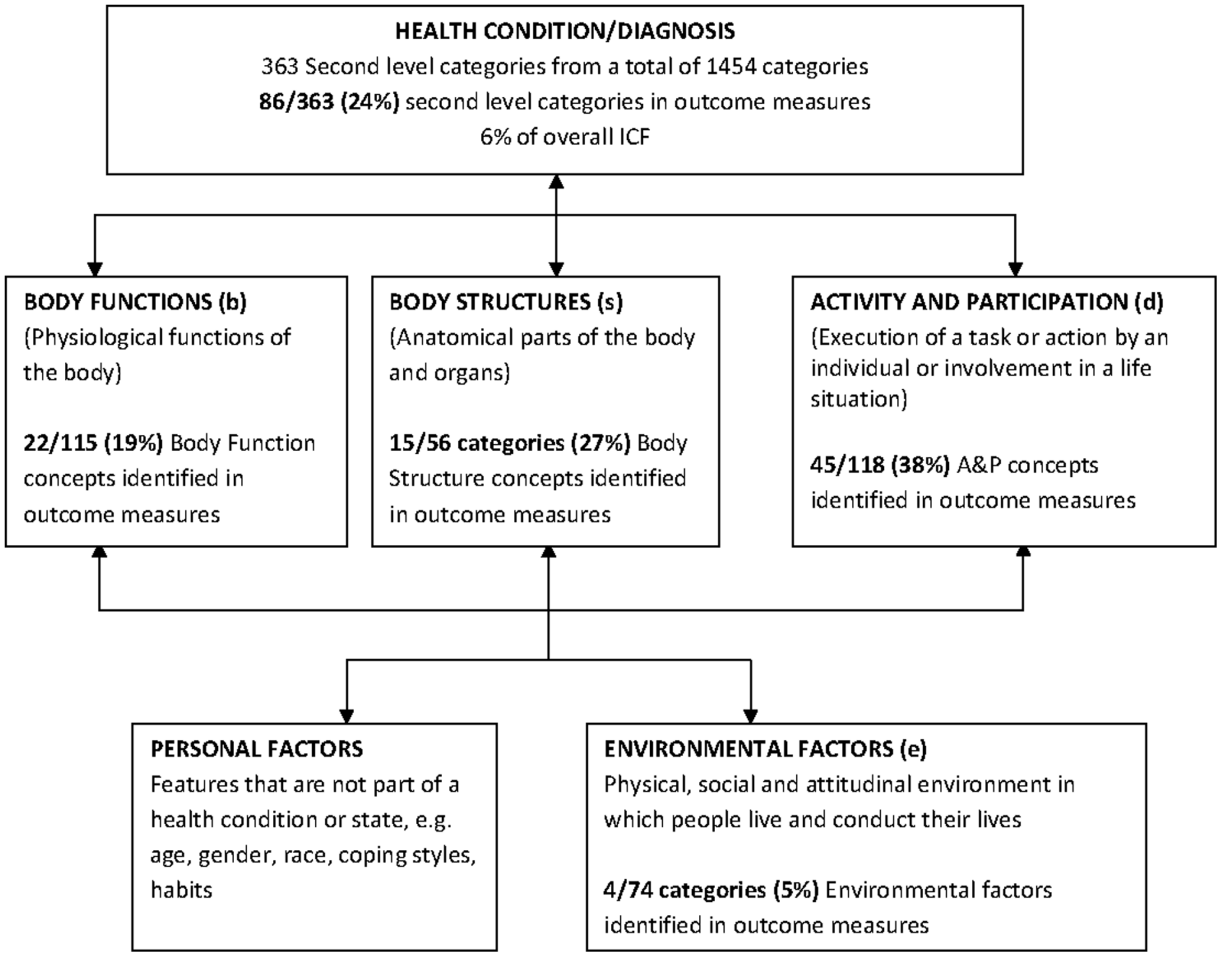
ICF framework and total number of second level ICF categories identified in six outcome measures.

Overall 250 meaningful concepts were identified across 132 items (questions) within six outcome measures (Table S5). The TOP captured the most meaningful concepts (n = 107), whilst the rest were distributed across the SF-36 (n = 53), FIM (n = 37), WHODAS ll (n = 34), EQ-5D (n = 10) and the GOS (n = 9). These 250 meaningful concepts were linked to 86 of a possible 363 second level categories (24%), and represented only 6% of the total number of ICF categories (n = 1454) (Figure 2).

Table 3 summarises the content analysis of the individual measures. Bandwidth was calculated in relation to 363 second level categories, rather than all 1454 ICF categories. The SF-36 was used approximately three times more (14 citations) than the other generic measures but represents a small proportion of the ICF (bandwidth of 6.3%). Items were linked to nine of the thirty ICF chapters (30%): two in the *body function* (b) component and seventeen in the *activity and participation* (d) component. The small content diversity (0.43) reflects the depth of the measure (several items and their concepts are dedicated to measure the same topic or ICF category).

The EQ5D was the second most cited measure. Both the EQ5D and the GOS are very concise measures consisting of only 5 items each. Their ICF representation in terms of bandwidth is very small, 2.8% and 2.5% respectively, but very specific in terms of which ICF categories they measure (content density  = 2 and 1.8 respectively). Despite the limited number of items the EQ5D represent eight ICF chapters (27%), two in the *body function* (b) component and six in the *activity and participation* (d) component.

The WHODAS II and the FIM were similar in their ICF representation with a respective bandwidth of 6.6% and 8.0%. Both measures covered nine chapters of the ICF with the WHODAS II containing eight categories in the *activity and participation* (d) component compared to five represented in the FIM.

The TOP was the only trauma specific measure, although cited only once [26]. The TOP had the largest ICF representation with 61 categories (bandwidth = 16.8%) and covered seventeen ICF chapters (57%). The TOP was the only measure to include items in the environmental factors component (n = 4).

### Overall representation of ICF categories contained in outcome measures

Only 18 (21%) of the 86 second level ICF categories occurred in more than three measures (Table S4). Fourteen of thirty possible chapters were represented by the outcome measures. Two *body function* chapters *mental functions* (b1) and *sensory functions of pain* (b2) were frequently represented (>30%). No measure contained categories relating to chapter 8 *functions of the skin and related structures* (b8). All nine *activity and participation* chapters were represented and linked to forty five ICF categories, with 27% (n = 32) second level categories frequently represented. Four *environmental factors* (e) were linked in one measure. Even administration of all outcome measures would result in poor assessment of the full breadth of the ICF.

## Discussion

The review provides an overview of how frequently used measures capture health outcome after injury using the ICF as a reference system. It is evident by the small number of measures identified here, that the evaluation of patient reported health outcome remains inconsistent and absent despite decades of trauma research. The most comprehensive measure captures less than 5% of possible health outcomes, and there were significant gaps in domains of the environment and activity and participation. Existing outcome measures do not describe the impact of major trauma on function, disability and health. We therefore do not fully understand the health outcomes of trauma patients.

Measuring the population burden of major trauma is a complex task, made particularly difficult by the heterogeneity of patient populations and injury patterns [61]. This task is made more problematic by the inconsistent use of outcome measures. Only five measures were used three times or more in studies included. Many of the outcome measures included in the analysis were generic. The SF-36 was cited most frequently and used in studies in the USA, Europe and the UK. This was not surprising as it is one of the most widely used generic measures to evaluate health related quality of life [31]. While the consistent application of the SF-36 could allow international comparison of trauma outcome, it captures only a small proportion of health outcomes. The measure with the greatest ICF representation (TOP) was cited only once [26] and also requires further validation studies [62]. Only one measure was developed within the ICF framework (WHODAS ll). Despite this the ICF representation is limited and important condition specific categories, such as the impact of scars and disfigurement are not captured. This large variation in measures, and the absence of functional tools in trauma registries [12] impedes comparison of outcome and an understanding of the impact of injury on different populations [63], [64].

Content analysis, confirmed that only a small proportion of health outcomes are captured by frequently used measures and there was little consistency across the measures in their coverage of the ICF. Existing outcome measures do not fully describe the impact of major trauma on function, health and disability. There must therefore be major gaps in our understanding of outcome after trauma.

The absence of a comprehensive health outcome measure in major trauma limits focused clinical care and research [65], [66]. The importance of this can be illustrated by the significant gaps we identified in the existing tools – such as in areas related to environmental factors and participation. People may have the physical ability to do their own shopping but are unable to leave the house to go shopping due to stairs at the front door. Their participation is restricted by environmental factors. Environmental factors such as education, access to medical insurance, trauma systems and support services has all been shown to impact on outcome [67], [68]. However, less than 2% of all environmental factors were captured with existing outcome measures used in trauma care. Similarly few measures truly capture return to work or the factors limiting return to work despite injury being the leading cause of death in working-aged adults [69]. Health outcomes cannot be assessed without an understanding of restrictions to participation and environmental barriers. Comprehensive assessment tools are required to improved service provision, clinical research and ultimately patient outcomes [70], [71].

The use of generic outcomes measures is not unique to trauma. However there is generally a stronger consensus in terms of which measurement to use in conditions such as stroke, brain injury and in multiple sclerosis [22], [72], [73]. In many ways, health outcome evaluation of these conditions is more advanced than in trauma studies. In recognition of the value of a standardised code based system for health outcome evaluation, ICF core sets exist for these and several other conditions [22], [73], [74]. This framework enables local and population-wide evaluation. The lack of an ICF-based framework for trauma limits outcome evaluation and understanding of the true health impact of injury.

## Limitations

The systematic review relied on a simplified review methodology, using specific rather than sensitive search strategies due to the heterogeneity of studies. Most included studies were observational in nature with only a few RCTs. However, the results reflect the current state of study design in trauma outcome studies. The majority of outcome measures included in the review and selected for mapping correspond with those recommended in previous trauma consensus papers [6], [17], [65], [75]. We do not discuss the *body structures component* (s) of the ICF in any depth and only one measure, the TOP, included *body structures*. *Body structure* categories describe the body part or location of the health impact rather than what the actual health impact which was the purpose of the review. Trauma scores such as the Injury Severity Score (ISS) [76] and the Abbreviated Injury Scale (AIS) [77] covers many aspects of *body structures* however they are used to determine mortality rather than morbidity and were excluded. Finally, we only included English articles which excluded literature that may have pertained to a better understanding of the Hannover Score for Polytrauma Outcome.

## Conclusion

Health outcomes after major trauma are not comprehensively described or captured due to limited outcome measures that assess only a small proportion of possible impacts. There is a strong need for further research towards a comprehensive outcome measure, based on patients' experiences. This should be developed within an internationally recognised framework that can fully describe and quantify the impact of injury on patients' health outcomes. The ICF represents a useful framework for future development of health outcomes instruments for trauma.

## Supporting Information

Table S1 **Search Strategy.**
(PDF)

Table S2 **Quality review of articles included.**
(PDF)

Table S3 **Studies included in the review.**
(PDF)

Table S4 **Frequently represented ICF chapters and categories linked in outcome measures.** Fourteen chapters and 45 second level ICF categories were frequently used in measures (relative frequency >30%). Of these, only 18 categories occurred in half of the measures.
(PDF)

Table S5 **Supporting information, ICF mapping.**
(PDF)

Checklist S1 (DOC)
