# Supplementary material for: Health Outcome after Major Trauma: What Are We Measuring?
Source: PLoS One. 2014 Jul 22;9(7):e103082. doi: 10.1371/journal.pone.0103082 (PMC4106876; doi:10.1371/journal.pone.0103082)
Supplement: Table S1 — Search Strategy. (PDF) [file pone.0103082.s001.pdf]

Table S1. Search Strategy

Search History

2. MEDLINE; exp WOUNDS AND INJURIES/; 646280 results.
1. MEDLINE; exp REHABILITATION/; 134865 results.
4. MEDLINE; 1 OR 3; 198828 results.
3. MEDLINE; rehabilitation.ti,ab; 89044 results.
5. MEDLINE; ("major trauma" OR "multiple injuries" OR "poly trauma").ti,ab; 4211 results.
7. MEDLINE; exp *"QUALITY OF LIFE"/; 44377 results.
8. MEDLINE; ("quality of life" OR "health" OR "wellbeing").ti,ab; 1009023 results.
9. MEDLINE; ("outcome measure" OR "outcome tool" OR "instrument").ti,ab [Limit to: Publication Year 2006-2012]; 44486 results.
6. MEDLINE; exp *MULTIPLE TRAUMA/; 6382 results.
10. MEDLINE; exp TREATMENT OUTCOME/ OR exp "OUTCOME ASSESSMENT (HEALTH CARE)"/; 592739 results.
12. MEDLINE; 7 OR 8; 1015515 results.
13. MEDLINE; 1 OR 3; 198828 results.
14. MEDLINE; 2 OR 5 OR 6; 647058 results.
15. MEDLINE; 11 AND 12 AND 13 AND 14 [Limit to: Publication Year 2006-2012]; 411 results.
11. MEDLINE; 9 OR 10 [Limit to: Publication Year 2006-2012]; 347987 results.
16. MEDLINE; 15 [Limit to: Publication Year 2006-2012 and (Age Groups All Adult 19 plus years) and (Languages English) and (Publication Types Clinical Trial, All or Clinical Trial, Phase I or Clinical Trial, Phase II or Clinical Trial, Phase III or Clinical Trial, Phase IV or Clinical Trial or Comparative Study or Consensus Development Conference or Consensus Development Conference, NIH or Controlled Clinical Trial or Evaluation Studies or Government Publications or Guideline or Interview or Meta Analysis or Multicenter Study or Practice Guideline or Randomized Controlled Trial or Research Support, American Recovery and Reinvestment Act or Research Support, NIH, Extramural or Research Support, NIH, Intramural or Research Support, Non US Gov't or Research Support, US Gov't, Non PHS or Research Support, US Gov't, PHS or Review or Scientific Integrity Review or Twin Study or Validation Studies)]; 237 results.
