# Supplementary material for: Health Outcome after Major Trauma: What Are We Measuring?
Source: PLoS One. 2014 Jul 22;9(7):e103082. doi: 10.1371/journal.pone.0103082 (PMC4106876; doi:10.1371/journal.pone.0103082)
Supplement: Table S2 — Quality review of articles included. (PDF) [file pone.0103082.s002.pdf]

Table S2. Quality review of articles included

Source	Study design, number of participants	Study objective	Standardised outcome instruments	Main findings	GRADE approach and comments
Ballabeni P; Burrus C; Luthi F; Gobelet C; Deriaz O (2011)	Prospective cohort study (*n=391)* Switzerland	To evaluate the association between recall of previous work environment and return to work (RTW) after hospitalisation in a rehabilitation hospital.	• Karasek's 31-item Job Content Questionnaire (JCQ)	People were less likely to return to work 1 year after hospital discharge if they perceived work to be of a higher physical demand. Social support at work was positively associated with return to work at all-time points.	**Quality rating =** Low<br>**Risk of bias:** Lack of blinding, **i**ndirectness (comparing different occupations) and inconsistent results (difference in population)<br>**Other bias:** Recall bias (self-report measures)
Baranyi *et al.*(2010)	Prospective cohort study (*n*=52*)* Germany	Evaluation of accident-related posttraumatic stress disorder (PTSD), and health-related quality of life.	• German version of the Clinician-Administered PTSD Scale (CAPS)<br>• Syndrom-Kurz Test (SKT)<br>• Beck Depression Inventory (BDI),<br>• Impact of Events Sale (IES)<br>• Dissociative Experience Scale (DES)<br>• Medical Outcomes Study Short Form Health Survey (SF-36)	Patients with poly-trauma, needs a biopsychosocial conceptual framework to reduce psychiatric morbidity following trauma surgery in general hospitals.	**Quality rating =** Low<br>**Risk of bias:** Inconsistent results due to small sample size and bias, prognostic imbalance.<br>**Other bias:** Study design, number of instruments used, loss to follow up.
Christensen MC; Banner C; Lefering R; Vallejo-Torres L; Morris S (2011)	Randomized control trial (n=347) Denmark	To determine risk factors of poor quality of life following traumatic injuries.	• Polytrauma Outcome Chart consisting of the<br>• Glasgow Outcome Scale (GOC)<br>• European Quality of Life Questionaire (EQ-5D)<br>• SF-36<br>• Trauma Outcome Profile	Demographic and socioeconomic characteristics as well and type of injury, and treatment received can predict quality of life.	**Quality rating =** Moderate<br>**Risk of bias:** >20% loss to follow up,<br>**Other bias:** Recall bias (self-report measures)

Author(s)	Study design, number of participants	Study objective	Standardised outcome instruments	Main findings	GRADE approach and comments
Derrett S, Davie G, Ameratunga S, Langley J. (2010)	Prospective cohort study (n=111) New Zealand	To evaluate the feasibility of collecting pre and post-injury data	• WHODAS II<br>• EQ5D	The recruitment and interview methods were both feasible and acceptable to participants.	**Quality rating** = Low<br>**Risk of bias:** >20% loss to follow up, prognostic imbalance, lack of internal controls.<br>**Other bias:** Recall bias (self-report measures)
Franzén C; Brulin C; Stenlund H; Björnstig U (2009)	Randomised control trial study (n=568) Sweden	To investigate whether nursing intervention via telephone follow-up can improve quality of life of injured road users.	• EQ-5D	Nursing intervention via telephone follow-up is effective at increasing the quality of life of injured road users. More research is needed to evaluate early interventions for recovery.	**Quality rating =** Low<br>**Risk of bias:** No allocation concealment. Unclear methods used for randomisation. No blinding<br>**Other bias:** Recall bias (self-report measures). >20% loss to follow up at 6 months
Gabbe BJ, Simpson PM, Sutherland AM, Wolfe R, Lyons RA, Cameron PA. (2013)	Prospective cohort study (n=617) Australia	To evaluate recovery after major trauma over a 24-months.	• SF-12<br>• Glasgow Outcome Scale-Extended (GOS-E)	There was a variation in the rate of recovery for some subgroups and different patterns of recovery. Time points for follow up and requires careful consideration.	**Quality rating =** Low<br>**Risk of bias:** >20% loss to follow up. prognostic imbalance, lack of internal controls.<br>**Other bias:** Study design, recall bias (self-report measures), publication bias

Author(s)	Study design, number of participants	Study objective	Standardised outcome instruments	Main findings	GRADE approach and comments
Gabbe BJ, Simpson PM, Sutherland AM, Wolfe R, Fitzgerald MC, Judson R, Cameron PA. (2012)	Retrospective database review (n=4986) Australia	To describe outcomes of major trauma survivors managed in an organized trauma system.	• Glasgow Outcome Scale-Extended (GOSE).	Cases managed at Major Trauma T Centres (level-1 trauma centres) demonstrated better functional outcomes which improved over time.	**Quality rating =** Low<br>**Risk of bias:** Study design, prognostic imbalance, lack of internal controls.<br>**Other bias:** Recall bias (self-report measures)
Gabbe BJ, Cameron PA, Hannaford AP, Sutherland AM, McNeil JJ. (2006).	Prospective cohort study (n=662) Australia	To provide a broad description of the long-term outcomes of major trauma patients and establish the follow-up rate of registry patients.	• Modified FIM	Patients can be followed up using a trauma registry although more sensitive outcome instruments are required	**Quality rating =** Low<br>**Risk of bias:** >20% loss to follow up.<br>**Other bias:** Study design, no blinding or allocation concealment, recall bias (self-report measures), insensitive outcome measure- modified FIM
Harris IA, Young JM, Rae H, Jalaludin BB, Solomon MJ. (2008)	Prospective cohort study (n=355) Australia	To explore potential predictors of general health after major physical trauma.	• SF-36	Worse physical outcomes often relates to injury compensation claims while improved physical outcome relates to time from injury and lower injury severity.	**Quality rating =** Low<br>**Risk of bias:** Reporting bias – inconsistent results after patient refusal and exclusion<br>**Other bias:** Study design, no blinding or allocation concealment, recall bias (self-report measures).
Holtslag HR; van Beeck EF; Lindeman E; Leenen LP (2007).	Prospective cohort study (n=335) The Netherlands	To describe the long-term functional consequences from major trauma.	• Glasgow Outcome Scale (GOS)<br>• EuroQol (EQ-5D)<br>Head injury symptom checklist (HISC).	The important independent predictors of long-term functional consequences after major trauma are injury localization, educational level.	**Quality rating =** Low<br>**Risk of bias:** No blinding, prognostic imbalance, lack of internal controls.<br>**Other bias:** Study design and dichotomising data

**Author(s)**	**Study design, number of participants**	**Study objective**	**Standardised outcome instruments**	**Main findings**	**GRADE approach and comments**
Holtslag H; Buskens E; Rommers C; Prevo A; van der Werken C (2006)	Prospective cohort study (n=186) The Netherlands	To measure functional recovery across several domains of daily living.	• Glasgow Outcome Scale (GOS)<br>• Groningen Activity Restriction Score (GARS)<br>• Sickness Impact Profile-136 (SIP)<br>• Short Form-36 (SF-36).	Functional outcome instruments do not accurately measure log term restrictions in the level of activities and participation. Mobility is severely restricted in lower extremity poly trauma patients for a significant period after injury.	**Quality rating =** Low<br>**Risk of bias:** Study design<br>**Other bias:** Loss to follow up, lack of internal controls. recall bias (self-report measures)
Jackson JC; Obremskey W; Bauer R; Greevy R; Cotton BA; Anderson V; Song Y; Ely EW (2007).	Prospective cohort study (n=58) USA	To determine the prevalence of emotional and functional difficulties as well as cognitive impairment in trauma patients in intensive without intracranial hemorrhage	• Informant Questionnaire of Cognitive Decline in the Elderly-Short Form (IQCODE-SF)<br>• SF-36<br>• Beck's Depression Inventory<br>• Katz Index of Independence in Activities of Daily Living. Activities<br>• Davidson Trauma Scale<br>• Beck's Anxiety Inventory<br>• Functional Activities Questionnaire (FAQ)<br>• Awareness questionnaire	Cognitive impairment is present in trauma patients with skull fractures, concussions and those without intra cranial haemorrhage. Poor quality of life, functional deficits and an inability to return to work resulted due to cognitive impairment.	**Quality rating =** Low<br>**Risk of bias:** Study design and self-complete questionnaires, **r**ecall bias<br>**Other bias:** Inconsistent results in terms of differences in patient population, imprecision – small sample size. Prognostic imbalance

Author(s)	Study design, number of participants	Study objective	Standardised outcome instruments	Main findings	GRADE approach and comments
Kiely JM; Brasel KJ; Weidner KL; Guse CE; Weigelt JA (2006)	Prospective cohort study (n=123) USA	To evaluate predictor of quality of life 6 months post injury.	• SF-36<br>• FIM<br>• PTSD Checklist (PCL)<br>• Centre for Epidemiologic Studies Depression Scale (**CES-D**-10)	Interventions should be targeted to improve physical functioning, reduce depression and post traumatic stress and provide adequate social support.	**Quality rating =** Low<br>**Risk of bias:** Study design, no blinding. Prognostic imbalance, lack of internal controls.<br>**Other bias:** Recall bias (self-report measures). Reporting bias >20% loss to follow up
Langley J, Derrett S, Davie G, Ameratunga S, Wyeth E (2011).	Prospective cohort study (*n=2856)* New Zealand	To evaluate short term functional outcomes following injury	• EQ-5D	Patients with minor injuries may have worse outcome due to pre-injury socio-demographic and health characteristics and should be included in trauma follow up.	**Quality rating =** Low<br>**Risk of bias:** Study design, no blinding, prognostic imbalance<br>**Other bias:** Recall bias (self-report measures).
Livingston DH; Tripp T; Biggs C; Lavery RF (2009)	Prospective cohort study (n=100)	To evaluate long term outcome of severe injury following injury following intensive care admission	• GOS<br>• FIM<br>• Modified FIM	Even at 3 years after severe sinjury people continue to have significant impairments including inability to return to work or regain previous levels of activity. Reintegration into society is limited.	**Quality rating =** Low<br>**Risk of bias:** Study design, dichotomising scores<br>**Other bias:** Reporting bias, >20% loss to follow up. Recall bias (self-report measures).
Mackenzie EJ; Rivara FP; Jurkovich GJ; Nathens AB; Egleston BL; Salkever DS; Frey KP; Scharfstein DO (2008).	Retrospective cohort study (n=1389) USA	To evaluate the effectiveness of trauma centres to improve functional outcomes, especially for patients with major lower-limb trauma	• SF-36<br>• Musculoskeletal Function Assessment (MFA) –mobility subscale<br>• Center for Epidemiologic Studies Depression Scale (CESD-R)	Patients who sustain high-energy lower-limb trauma benefit from treatment at a level-I trauma centre.	**Quality rating =** Low<br>**Risk of bias:** Study design, dichotomising scores, prognostic imbalance, lack of internal controls.<br>**Other bias:** Reporting bias. Recall bias (self-report measures).

Author(s)	Study design, number of participants	Study objective	Standardised outcome instruments	Main findings	GRADE approach and comments
Orwelius L.,Bergkvist M.,Nordlund A.,Simonsson E.,Nordlund P.,Backman C.,Sjoberg F. (2012)	Prospective multicenter study (n=108) Sweden	To investigate HRQoL after trauma and specifically evaluate the impact of ICU-related, socio demographic factors and pre existing disease,	• SF-36	Quality of life is reduced at 2 years, especially physiological recovery likely due to pre-existing disease.	**Quality rating =** Low<br>**Risk of bias:** Study design, lack if internal controls.<br>**Other bias:** Reporting bias. Recall bias (self-report measures). Reporting bias >20% loss to follow up.
Pape HC, Probst C, Lohse R, Zelle BA, Panzica M, Stalp M, Steel JL, Duhme HM, Pfeifer R, Krettek C, Sittaro NA (2010).	Prospective cohort study (n=637) Germany	To evaluate if reduced functional and psychosocial outcome can be contributed to certain injury patterns.	• SF-12<br>• Hannover Score for Poly-trauma Outcome, HASPOC	Traumatic lower extremity amputation, initial abbreviated injury scale score and spinal injuries result in worse outcome at ten year follow up.	**Quality rating =** Low<br>**Risk of bias:** prognostic imbalance, lack of internal controls.<br>**Other bias:** Recall bias (self-report measures). Reporting bias >20% loss to follow up. Questionable validity and reliability of HASPOC tool.
Pirente N.; Blum C.; Wortberg S.; Bostanci S.; Berger E.; Lefering R.; Bouillon B.; Rehm K.E.; Neugebauer E.A.M. (2007)	Randomised control trial (n=171) Germany	To evaluate if early cognitive behavioural therapy will improve quality of life if multi-trauma patients.	• Beck's Depression Inventory (BDI)<br>• SF-36<br>• State-Trait Anxiety Inventory (STAI)<br>• Symptom Checklist 90-Revised (SCL 90R)<br>• Social support Questionnaire (Fragebogen zur Sozialen Unterstützung; F-SOZU-22)	Cognitive therapy is not effective in improving overall HRQOL of severely injured patients early on although it shows promising effects on anxiety and depression at 12 months.	**Quality rating =** Low<br>**Risk of bias:** No blinding of research therapist<br>**Other bias:** 23% of randomised patients withdrew. Recall bias (self-report measures). >20% loss to follow up at 12 months. Groups not matched despite randomisation.

Author(s)	Study design, number of participants	Study objective	Standardised outcome instruments	Main findings	GRADE approach and comments
Polinder S; van Beeck EF; Essink-Bot ML; Toet H; Looman CW; Mulder S; Meerding WJ (2007)	Prospective cohort study (n=3231) The Netherlands	To compare functional recovery from injury for non hospitalised and hospitalised patients.	• EQ-5D	Injured patients that are hospitalised take substantially longer to recover when compared to the 5 months recovery period of non-hospitalised patients. .	**Quality rating =** Low<br>**Risk of bias:** Missing internal controls.<br>**Other bias:** > 50% loss to follow up. Different group comparison
Probst C, Zelle B, Panzica M, Lohse R, Sitarro NA, Krettek C, Pape HC (2010).	Longitudinal cohort study (n=637) Germany	To evaluate if there is a difference in outcome following trauma between genders.	• Hannover Score for Poly-trauma Outcome<br>• Short form-12, HADS	Men suffer less psychological impairment than woman with similar injuries.	**Quality rating =** Low<br>**Risk of bias:** Failure to control for confounding factors<br>**Other bias:** Time since injury, measures used, difference in ample size, > 25% loss to follow up
Ringburg AN, Polinder S, van Ierland MC, Steyerberg EW, van Lieshout EM, Patka P, van Beeck EF, Schipper IB (2011).	Prospective cohort study (n=246) The Netherlands	Assess the health-related quality of life of survivors of severe trauma 1 year after injury	• Health Utilities Index (HUI)<br>• EQ-5D	At 12 months after a traumatic injury, people continue to have poor functional outcome and quality of life.	**Quality rating =** Moderate<br>**Risk of bias:** Study design but good tools and multivariate analysis<br>**Other bias**:>30% loss to follow up,

Author(s)	Study design, number of participants	Study objective	Standardised outcome instruments	Main findings	GRADE approach and comments
Sayer NA; Chiros CE; Sigford B; Scott S; Clothier B; Pickett T; Lew HL (2008).	Retrospective cohort study (n=188) USA	To describe rehabilitation outcomes among patients who sustain blast and other injuries in Iraq and Afghanistan.	• Functional Independence Measure (FIM)	There is an ongoing need for better assessment and treatment of pain and mental health problems among patients with polytrauma and blast-related injuries.	**Quality rating** = Low. **Risk of bias**: No allocation concealment and blinding. Prognostic imbalance, lack of internal controls. **Other bias:** study design, intervention not clearly described, no sample size calculation, use of the FIM which has ceiling effect, no other disease burden or QoL measurements used, retrospective chart review (reporting bias).
Schwartz I, Tsenter J, Shochina M, Shiri S, Kedary M, Katz-Leurer M, Meiner Z (2007)	Retrospective cohort study (n=72) Israel	Comparison of rehabilitation outcomes of multi-trauma terror victims versus non-terror victims.	• Functional Independence Measure (FIM)<br>• Impact of Events Scale (IES)	The reintegration and functional recovery of both groups are similar despite longer rehabilitation periods of terror victims. Both groups returned to previous occupation at a similar rate despite the terror group having higher levels of post traumatic stress.	**Quality rating** = Low. **Risk of bias**: No internal controls and prognostic imbalance. **Other bias:** study design, intervention not clearly described, no sample size calculation, use of the FIM which has ceiling effect,
Siddharthan K, Scott S, Bass E, Nelson A (2008).	Retrospective cohort study (n=116) USA	To evaluate rehabilitation outcome of people with military service-related injuries	• FIM	Pain management is essential to improve disability. Care co-ordination ensures optimal rehabilitation.	**Quality rating =** Very low **Risk of bias:** Study design, methodology, missing internal controls **Other bias:** 50% loss to follow up, reporting bias, no control for confounding factors

Author(s)	Study design, number of participants	Study objective	Standardised outcome instruments	Main findings	GRADE approach and comments
Soberg HL, Finset A, Bautz-Holter E, Sandvik L, Roise O (2007).	Prospective cohort study (n=100) Norway	To examine return to work and factors that predicted this for patients with severe multiple injuries.	• Brief Approach/Avoidance Coping Questionnaire<br>• Multidimensional Health Locus of Control<br>• Short Form-36<br>• World Health Organization Disability Assessment Schedule II (WHODAS-ll)	Many patients had not returned to work at 2 years due to social functioning and injury related factors.	**Quality rating** = Low.<br>**Risk of bias**: No allocation concealment, internal controls missing<br>**Other bias Bias**: study design, use of self-report measures (recall bias); time for follow up and questionable validity and reliability of outcome measures used.
Soberg HL, Bautz-Holter E, Roise O, Finset A(2007)	Prospective cohort study (n=105) Norway	To assess functioning and quality of life after severe injuries.	• Short Form (SF)-36<br>• World Health Organization Disability Assessment Schedule II (WHODAS II)	Most life domains are affected by long-lasting functional problems after multiple traumatic injuries.	**Quality rating** = Low.<br>**Risk of bias**: No sequence generation, allocation concealment and blinding.<br>**Other bias Bias**: study design, use of self-report measures (recall bias); intervention not defined.
Soberg HL, Bautz-Holter E, Roise O, Finset A (2010)	Prospective cohort study (n=99) Norway	To describe mental health and posttraumatic stress symptoms (PTSS) for patients with severe multiple trauma 2 years after injury.	• SF-36<br>• Post-Traumatic Symptom Scale 10 (PTSS-10)	Post traumatic stress symptoms were still present in 20 % of patients at 2 years.	**Quality rating =** Low<br>**Risk of bias:** No allocation concealment, prognostic imbalance.<br>**Other bias:** No comparative group, reason in regards to high variances. Use of self report measures and time since injury (recall bias)

Author(s)	Study design, number of participants	Study objective	Standardised outcome instruments	Main findings	GRADE approach and comments
Soberg H.L.,Finset A.,Roise O.,Bautz-Holter E. (2012)	Prospective, longitudinal cohort study (n=105) Norway	To describe the recovery of physical and mental health for people with multiple traumatic injuries from injury to 5 year follow up and examine predictors of recovery.	• SF-36<br>• World Health Organization Disability Assessment Schedule II (WHODAS II)	Despite improvement in physical and mental health, figures remained below population norms at 5 years. Health outcomes were dependent on personal and injury-related factors.	**Quality rating =** Low<br>**Risk of bias:** No allocation concealment, prognostic imbalance.<br>**Other bias:** No comparative group, reason in regards to high variances. Use of self report measures and time since injury (recall bias)
Steel J, Youssef M, Pfeifer R, Ramirez JM, Probst C, Sellei R, Zelle BA, Sittaro NA, Khalifa F, Pape HC (2010).	Prospective longitudinal cohort study (n=620) USA	To evaluate long-term consequences of multiple blunt force trauma.	• SF-12,	A broader range of sensitive measures are needed to prospectively capture consequences of brain injury. Evidence-based rehabilitation interventions are required to improve physical and psychological outcome.	**Quality rating =** Low<br>**Risk of bias:** Prognostic imbalance, different measures of exposure/outcome.<br>**Other bias:** Self-report measures (recall bias); time for follow up (10 years), ++ loss to follow up, patient population majority brain injury (skewed population sample)
Sutherland AG; Alexander DA; Hutchison JD (2006)	Prospective cohort study (n=200) UK	To investigated the relationship between physical and psychological recovery after musculoskeletal trauma	• General Health Questionnaire (GHQ)<br>• Sickness Impact Profile (SIP)<br>• Musculoskeletal Function Assessment (MFA)<br>• SF-36	The strong correlation between impaired functional outcome and psychological recovery after musculoskeletal trauma.	**Quality rating =** Low<br>**Risk of bias:** Failure to adequately control for confounding factors<br>**Other bias**: > 20% loss to follow up, recall bias (self-complete questionnaires). Questionable validity of outcome measure (MAF)

Author(s)	Study design, number of participants	Study objective	Standardised outcome instruments	Main findings	GRADE approach and comments
Sutherland AG; Suttie S; Alexander DA; Hutchison JD (2011).	Prospective cohort study (n=104) UK	To investigate the long term recovery from post-traumatic psychopathology.	• General Health Questionnaire (GHQ)<br>• Sickness Impact Profile (SIP)<br>• Musculoskeletal Function Assessment (MFA)<br>• SF-36	There is a strong relationship between ongoing psychological consequences and poor physical recovery while injury severity did not play an important role in psychological recovery.	**Quality rating =** Low<br>**Risk of bias:** Failure to adequately control for confounding factors.<br>**Other bias:** : > 48% loss to follow up, recall bias (self-complete questionnaires)
Van Aswegen H, Myezwa H, Mudzi W, Becker P (2011)	Prospective cohort study (n=42) South Africa	To evaluate quality of life of survivors of penetrating trauma in South Africa	• SF-36	Patients continued to have poor quality of life at 6 months when compared to population norms	**Quality rating =** Low<br>**Risk of bias:** No internal controls and prognostic imbalance.<br>**Other bias:** Reporting bias >20% follow up. Recall bias (self-report measures).
Zeckey, Christian, Hildebrand, Frank, Pape, Hans-Christoph, Mommsen, Philipp, Panzica, Martin, Zelle, Boris A., Alexander Sittaro, Nicola, Lohse, Ralf, Krettek, Christian, Probst, Christian (2011).	Prospective longitudinal cohort study (n=620) Germany	To evaluate the difference in outcome of poly trauma patients with and without head injuries.	• Hannover Score for Polytrauma Outcome (HASPOC)<br>• Short Form 12 (SF-12)<br>• Glasgow Outcome Scale (GOS)	When matched for injury severity and gender, poly trauma patients with head injured have worse outcome than patients without head injuries.	**Quality rating =** Low<br>**Risk of bias:** Recruitment not clear, recall bias (self report measures). Prognostic imbalance<br>**Other bias:** Questionable validity of outcome measure - Hannover Score for Poly trauma Outcome (HASPOC), no allocation concealment. >50% loss to follow up.
