# Supplementary material for: Health Outcome after Major Trauma: What Are We Measuring?
Source: PLoS One. 2014 Jul 22;9(7):e103082. doi: 10.1371/journal.pone.0103082 (PMC4106876; doi:10.1371/journal.pone.0103082)
Supplement: Table S3 — Studies included in the review. (PDF) [file pone.0103082.s003.pdf]

Table S3. Studies included in the review

Source	Study design	Number of participants	Country	Standardised outcome instruments
Ballabeni et al, 2011 [53]	Prospective cohort	391	Switzerland	Karasek's 31-item Job Content Questionnaire (JCQ)
Baranyi et al, 2010 [45]	Prospective cohort	52	Germany	German version of the Clinician-Administered PTSD Scale (CAPS)<br>Syndrom-Kurz Test (SKT)<br>Beck Depression Inventory (BDI),<br>Impact of Events Sale (IES)<br>Dissociative Experience Scale (DES)<br>Medical Outcomes Study Short Form Health Survey (SF-36)
Christensen et al, 2011 [26]	RCT	347	Denmark	Polytrauma Outcome Chart consisting of the -<br>Glasgow Outcome Scale (GOC)<br>European Quality of Life Questionnaire (EQ-5D)<br>SF-36<br>Trauma Outcome Profile (TOP)
Derrett et al, 2010 [40]	Prospective cohort	111	New Zealand	World Health Organisation Disability Assessment Schedule II (WHODAS II)<br>EQ-5D
Franzén et al, 2009 [28]	RCT	568	Sweden	EQ-5D
Gabbe et al, 2013 [39]	Prospective cohort	617	Australia	Study Short Form 12 (SF-12)<br>Glasgow Outcome Scale-Extended (GOS-E)
Gabbe et al, 2012 [38]	Database review	4986	Australia	GOS-E
Gabbe et al,2006 [37]	Prospective cohort	662	Australia	Modified Functional Independence Measure (FIM)
Harris et al, 2008 [36]	Prospective cohort	355	Australia	SF-36
Holtslag et al, 2007 [47]	Prospective cohort	335	The Netherlands	Glasgow Outcome Scale (GOS)<br>EuroQol (EQ-5D)<br>Head injury symptom checklist (HISC)
Holtslag et al, 2006 [46]	Prospective cohort	186	The Netherlands	Glasgow Outcome Scale (GOS)<br>Groningen Activity Restriction Score (GARS)<br>Sickness Impact Profile-136 (SIP)<br>SF-36

Jackson et al, 2007 [30]	Prospective cohort	58	USA	Informant Questionnaire of Cognitive Decline in the Elderly-Short Form (IQCODE-SF)<br>SF-36<br>Beck's Depression Inventory (BDI)<br>Katz Index of Independence in Activities of Daily Living<br>Davidson Trauma Scale (DTS)<br>Beck's Anxiety Inventory<br>Functional Activities Questionnaire ((FAQ)<br>Awareness questionnaire
Kiely et al, 2006 [31]	Prospective cohort	123	USA	SF-36<br>FIM<br>Post-Traumatic Stress Disorder Checklist (PCL)<br>Centre for Epidemiologic Studies Depression Scale (CES-D-10)
Langley et al, 2011 [41]	Prospective cohort	2856	New Zealand	EQ-5D
Livingston et al, 2009 [32]	Prospective cohort	100	USA	GOS<br>FIM<br>Modified FIM
Mackenzie et al, 2008 [33]	Retrospective cohort	1389	USA	SF-36<br>Musculoskeletal Function Assessment (MFA) –mobility subscale<br>Centre for Epidémiologique Studies Depression Scale (CESD-R)
Orwelius et al, 2012 [52]	Prospective cohort	108	Sweden	SF-36
Pape et al, 2010 [44]	Prospective cohort	637	Germany	SF-12<br>Hannover Score for Poly-trauma Outcome (HASPOC)
Pirente et al, 2007 [27]	RCT	171	Germany	Beck's Depression Inventory (BDI)<br>SF-36<br>State-Trait Anxiety Inventory (STAI)<br>Symptom Checklist 90-Revised (SCL 90R)<br>Social support Questionnaire (Fragebogen zur Sozialen Unterstützung; F-SOZU-22)
Polinder et al, 2007 [48]	Prospective cohort	3231	The Netherlands	EQ-5D
Probst et al, 2010 [42]	Prospective cohort	637	Germany	Hannover Score for Poly-trauma Outcome<br>Short form-12, HADS

Ringburg et al, 2011 [49]	Prospective cohort	246	The Netherlands	Health Utilities Index (HUI)<br>EQ5D
Sayer et al, 2008 [34]	Retrospective cohort	188	USA	Functional Independence Measure (FIM)
Schwartz et al, 2007 [54]	Retrospective cohort	72	Israel	Functional Independence Measure (FIM)<br>Impact of Events Scale (IES)
Siddharthan et al, 2008 [35]	Retrospective cohort	116	USA	FIM
Soberg et al, 2007 [50]	Prospective cohort	100	Norway	Brief Approach/Avoidance Coping Questionnaire<br>Multidimensional Health Locus of Control<br>Short Form-36<br>WHODAS-ll
Soberg et al, 2007 [8]	Prospective cohort	105	Norway	Short Form (SF)-36<br>WHODAS II
Soberg et al, 2010 [51]	Prospective cohort	99	Norway	SF-36<br>Post-Traumatic Symptom Scale 10 (PTSS-10)
Soberg et al, 2012 [9]	Prospective cohort	105	Norway	SF-36<br>WHODAS II
Steel et al, 2010 [29]	Prospective cohort	620	USA	SF-12
Sutherland et al, 2006 [24]	Prospective cohort	200	UK	General Health Questionnaire (GHQ)<br>Sickness Impact Profile (SIP)<br>Musculoskeletal Function Assessment (MFA)<br>SF-36
Sutherland et al, 2011 [25]	Prospective cohort	104	UK	General Health Questionnaire (GHQ)<br>Sickness Impact Profile (SIP)<br>Musculoskeletal Function Assessment (MFA)<br>SF-36
Van Aswegen et al, 2011 [55]	Prospective cohort	42	South Africa	SF-36
Zeckey et al, 2011 [43]	Prospective cohort	620	Germany	HASPOC<br>SF-12<br>Glasgow Outcome Scale (GOS)
