# Supplementary material for: Health Outcome after Major Trauma: What Are We Measuring?
Source: PLoS One. 2014 Jul 22;9(7):e103082. doi: 10.1371/journal.pone.0103082 (PMC4106876; doi:10.1371/journal.pone.0103082)
Supplement: Table S4 — Frequently represented ICF chapters and categories linked in outcome measures. Fourteen chapters and 45 second level ICF categories were frequently used in measures (relative frequency >30%). Of these, only 18 categories occurred in half of the measures. (PDF) [file pone.0103082.s004.pdf]

Table S4. Frequently represented ICF chapters and categories linked in outcome measures

ICF

BODY FUNCTIONS (b) n=10	%
**CHAPTER 1: Mental functions (b1)**	
b117 Intelectual functions	33
b126 Temprament and personality functions	33
b130 Energy and drive	33
b134 Sleep functions	33
b 140 Attention functions	33
b 144 Memory functions	50
b152 Emotional functions	67
b160 Thought functions	50
b180 Experience of self and time functions	33
**CHAPTER 2: Sensory functions and pain (b2)**	
b280 Sensation of pain	50

ACTIVITYAND PARTICIPATION (d) n=32	%
**CHAPTER 1: Learning and applying knowledge (d1)**	
d160 Focusing attention	33
d175 Solving problems	50
**CHAPTER 2: General tasks and demands (d2)**	
d230 Carrying out daily routine	33
d240 Handling stress	33
**CHAPTER 3: Communication (d3)**	
d310 Communication	33
d350 Conversation	33
**CHAPTER 4: Mobility (d4)**	
d410 Changing basic body position	50
d415 Maintaining a body position	33
d450 Walking	67
d455 Moving around	33
d465 Moving around using equipment	33
**CHAPTER 5: Self-care (d5)**	
d510 Washing oneself	83
d520 Caring for body parts	33
d530 Toiletting	33
d540 Dressing	83
d550 Eating	50
d560 Drinking	33
d570 Looking after one's health	33
**CHAPTER 6: Domestic life (d6)**	
d640 Doing housework	83
**CHAPTER 7: Interpersonal interaction (d7)**	
d710 Basic Interpersonal interaction	50
d720 Complex interpersonal interaction	33
d750 Informal social relationships	50
d760 Family relationships	67
d770 Intimate relationships	50
**CHAPTER 8: Major life areas (d8)**	
d820 School education	33
d825 Vocational training	33
d830 Higher education	33
d845 Acquiring, and keeping a job	33
d850 Remunerative employment	67
d870 Economic self-sufficiency	33
**CHAPTER 9: Community, Social and civic life (d9)**	
d910 Community life	50
d920 Recreation and leisure	67

ENVIRONMENTAL FUNCTIONS (e) n=3	%
**CHAPTER 1: Products and technology (e1)**	
e115 Products/technology for daily living	33
**CHAPTER 3: Support and relationships (e3)**	
e310 Immediate family	33
**CHAPTER 4: Attitudes (e4)**	
e410 Individual attitudes of family	33

Table S4. Fourteen chapters and 45 second level ICF categories were frequently used in measures (relative frequency >30%). Of these, only 18 categories occurred in half of the measures.
