# Supplementary material for: Health Outcome after Major Trauma: What Are We Measuring?
Source: PLoS One. 2014 Jul 22;9(7):e103082. doi: 10.1371/journal.pone.0103082 (PMC4106876; doi:10.1371/journal.pone.0103082)

Table S5: Supporting information ICF mapping

	SF-36	EQ-5D	FIM	GOS	WHODAS II	TOP	Absolute frequency (n)	Absolute frequency%	Relative frequency n	Relative frequency%
Number of studies cited in	14	7	5	5	4	1				
Number of items in measure	36	5	18	5	36	32				
Total concepts per measure	53	10	37	9	34	107				
ICF categories	23	10	29	9	24	61				
**Body Function (b) n=22**	6	2	10	2	3	14				
**CHAPTER 1: Mental functions (b1)**										
b110 Consciousness functions				1			1	17%	1	17%
b117 Intelectual functions			1	1			2	33%	2	33%
b126 Temprament and personality functions					1	2	3	50%	2	33%
b130 Energy and drive	4					3	7	117%	2	33%
b134 Sleep functions	1					1	2	33%	2	33%
b 140 Attention functions			1			3	4	67%	2	33%
b 144 Memory functions			1		1	2	4	67%	3	50%
b147 Psychomotor functions						2	2	33%	1	17%
b152 Emotional functions	5	1			1	7	14	233%	4	67%
b160 Thought functions	1		1			2	4	67%	3	50%
b164 Higher-level cognitive functions			2				2	33%	1	17%
b167 Mental function of language			1				1	17%	1	17%
b180 Experience of self and time functions	1					4	5	83%	2	33%
**CHAPTER 2: Sensory functions and pain (b2)**										
b280 Sensation of pain	2	1				14	17	283%	3	50%
**CHAPTER 3:**										
b330 Fluency and rhythm of speech functions			1				1	17%	1	17%
**CHAPTER 4: Functions of the cardiovascular, haematological, immunological and respiratory systems**										

b410 Heart functions						1	1	17%	1	17%
b455 Exercise tolerance functions						1	1	17%	1	17%
	**SF-36**	**EQ-5D**	**FIM**	**GOS**	**WHODAS II**	**TOP**	**Absolute frequency (n)**	**Absolute frequency%**	**Relative frequency n**	**Relative frequency%**
**CHAPTER 5: Functions of the digestive, metabolic and endocrine systems**										
b525 Defecation functions			1				1	17%	1	17%
b550 Thermoregulatory functions						1	1	17%	1	17%
**CHAPTER 6: Genitourinary and reproductive functions**										
b610 Urination functions			1				1	17%	1	17%
b639 Urinary functions, other specified and unspecified			1				1	17%	1	17%
**CHAPTER 7: Neuro musculoskeletal and movement-related functions**										
b735 Muscle tone functions						1	1	17%	1	17%
**Body Structure (s) n=15**	0	0	0	1	0	14				
s110 Structure of brain				1			1	17%	1	17%
14 body structures mentioned in TOP						14	14	233%	1	17%
**Activity and Participation (d) n=45**	17	8	19	6	21	29				
**CHAPTER 1: Learning and applying knowledge**										
d155 Acquiring a skill					2		2	33%	1	17%
d160 Focusing attention					1	2	3	50%	2	33%
d163 Thinking						2	2	33%	1	17%
d175 Solving problems			1		1	2	4	67%	3	50%
**CHAPTER 2: General tasks and demands**										
d220 Undertaking multiple tasks	1						1	17%	1	17%
d230 Carrying out daily routine	2					2	4	67%	2	33%
d240 Handling stress and other psychological demands	1					5	6	100%	2	33%

	SF-36	EQ-5D	FIM	GOS	WHODAS II	TOP	Absolute frequency (n)	Absolute frequency%	Relative frequency n	Relative frequency%
**CHAPTER 3: Communication**										
d310 Communicating with – receiving – spoken messages			1		1		2	33%	2	33%
d315 Communicating with – receiving – nonverbal messages			1				1	17%	1	17%
d335 Producing non verbal messages			1				1	17%	1	17%
d350 Conversation			1		2		3	50%	2	33%
**CHAPTER 4: Mobility**										
d410 Changing basic body position	1		2		1		4	67%	3	50%
d415 Maintaining a body position					1	1	2	33%	2	33%
d420 Transferring oneself			3				3	50%	1	17%
d430 Lifting and carrying objects	3						3	50%	1	17%
d450 Walking	3	1	1		1		6	100%	4	67%
d455 Moving around	6		3				9	150%	2	33%
d460 Moving around in different locations					2		2	33%	1	17%
d465 Moving around using equipment			1			1	2	33%	2	33%
**CHAPTER 5: Self-care**										
d510 Washing oneself	1	1	1		1	1	5	83%	5	83%
d520 Caring for body parts			2			1	3	50%	2	33%
d530 Toiletting			1			1	2	33%	2	33%
d540 Dressing	1	1	2		1	1	6	100%	5	83%
d550 Eating			1		1	1	3	50%	3	50%
d560 Drinking			1			1	2	33%	2	33%
d570 Looking after one's health	6					3	9	150%	2	33%
**CHAPTER 6: Domestic life**										
d640 Doing housework	3	1		1	2	1	8	133%	5	83%

	SF-36	EQ-5D	FIM	GOS	WHODAS II	TOP	Absolute frequency (n)	Absolute frequency%	Relative frequency n	Relative frequency%
**CHAPTER 7: Interpersonal interactions and relationships**										
d710 Basic Interpersonal interaction	1		1			1	3	50%	3	50%
d720 Complex interpersonal interaction					1	1	2	33%	2	33%
d730 Relating with strangers					1		1	17%	1	17%
d750 Informal social relationships	1				2	1	4	67%	3	50%
d760 Family relationships	1	1	1			1	4	67%	4	67%
d770 Intimate relationships			1		1	3	5	83%	3	50%
d799 Interpersonal interaction and relationships, unspecified						1	1	17%	1	17%
**CHAPTER 8: Major life areas**										
d820 School education		1				1	2	33%	2	33%
d825 Vocational training				1		1	2	33%	2	33%
d830 Higher education				1		1	2	33%	2	33%
d845 Acquiring, keeping and terminating a job				1	3		4	67%	2	33%
d850 Remunerative employment	4	1		1		2	8	133%	4	67%
d855 Non-renumerative employment						1	1	17%	1	17%
d860 Basic economic transactions						1	1	17%	1	17%
d870 Economic self-sufficiency					1	1	2	33%	2	33%
**CHAPTER 9: Community, social and civic life**										
d910 Community life	1			1	3		5	83%	3	50%
d920 Recreation and leisure	3	1			1	1	6	100%	4	67%
d930 Religion and spirituality					1		1	17%	1	17%

	SF-36	EQ-5D	FIM	GOS	WHODAS II	TOP	Absolute frequency (n)	Absolute frequency%	Relative frequency n	Relative frequency%
**Environment (e) n=4**	0	0	0	0	0	4				
**CHAPTER 1 :Products and technology**										
e115 Products and technology for personal daily living						2	2	33%	1	17%
**CHAPTER 3: Support and relationships**										
e310 Immediate family						2	2	33%	1	17%
**CHAPTER 4: Attitudes**										
e410 Individual attitudes of immediate family						2	2	33%	1	17%
**CHAPTER 5: Services, systems and policies**										
e590 Labour and employment services						1	1	17%	1	17%

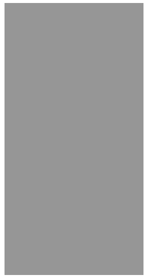

Supplement: Table S5 — Supporting information, ICF mapping. (PDF) [file pone.0103082.s005.pdf]
