# Supplementary material for: Health Outcome after Major Trauma: What Are We Measuring?
Source: PLoS One. 2014 Jul 22;9(7):e103082. doi: 10.1371/journal.pone.0103082 (PMC4106876; doi:10.1371/journal.pone.0103082)
Supplement: Checklist S1 — (DOC) [file pone.0103082.s006.doc]

# Title: Health outcome after major trauma: What are we measuring?

**Authors:** Karen Hoffman^a^, Elaine Cole^a^, E. Diane Playford^b^, Eva Grill^c^, Helene L. Soberg ^d^, Karim Brohi^a^

| **Section/topic** | **#** | **Checklist item** | **Reported on page #** |
| --- | --- | --- | --- |
| **TITLE** | | |  |
| Title | 1 | Identify the report as a systematic review, meta-analysis, or both. | Background and introduction; Methods, results and Table S2. Quality review of articles included |
| **ABSTRACT** | | |  |
| Structured summary | 2 | Provide a structured summary including, as applicable: background; objectives; data sources; study eligibility criteria, participants, and interventions; study appraisal and synthesis methods; results; limitations; conclusions and implications of key findings; systematic review registration number. | Background and introduction; Methods and Table S2. Quality review of articles included |
| **INTRODUCTION** | | |  |
| Rationale | 3 | Describe the rationale for the review in the context of what is already known. | Background and introduction |
| Objectives | 4 | Provide an explicit statement of questions being addressed with reference to participants, interventions, comparisons, outcomes, and study design (PICOS). | Background and introduction |
| **METHODS** | | |  |
| Protocol and registration | 5 | Indicate if a review protocol exists, if and where it can be accessed (e.g., Web address), and, if available, provide registration information including registration number. | n/a |
| Eligibility criteria | 6 | Specify study characteristics (e.g., PICOS, length of follow-up) and report characteristics (e.g., years considered, language, publication status) used as criteria for eligibility, giving rationale. | Study selection and inclusion/exclusion criteria |
| Information sources | 7 | Describe all information sources (e.g., databases with dates of coverage, contact with study authors to identify additional studies) in the search and date last searched. | Data sources and search strategy, Table S1 and Figure 1 |
| Search | 8 | Present full electronic search strategy for at least one database, including any limits used, such that it could be repeated. | Table S1. Search Strategy |
| Study selection | 9 | State the process for selecting studies (i.e., screening, eligibility, included in systematic review, and, if applicable, included in the meta-analysis). | Study selection and inclusion/exclusion criteria; Screening and data extraction |
| Data collection process | 10 | Describe method of data extraction from reports (e.g., piloted forms, independently, in duplicate) and any processes for obtaining and confirming data from investigators. | Figure 1, Screening and data extraction, ICF content analysis |
| Data items | 11 | List and define all variables for which data were sought (e.g., PICOS, funding sources) and any assumptions and simplifications made. | Country of publication, study design, sample size, outcomes and outcome measures used. Please also see study selection and inclusion/exclusion criteria, Table S2 Quality review of articles included |
| Risk of bias in individual studies | 12 | Describe methods used for assessing risk of bias of individual studies (including specification of whether this was done at the study or outcome level), and how this information is to be used in any data synthesis. | Table S2. Quality review of articles included; |
| Summary measures | 13 | State the principal summary measures (e.g., risk ratio, difference in means). | Table S2. Quality review of articles included; ICF content analysis |
| Synthesis of results | 14 | Describe the methods of handling data and combining results of studies, if done, including measures of consistency (e.g., I^2^) for each meta-analysis. | Results were not synthesised as further analysis were done. ICF content analysis; Table S2. Quality review of articles included |

| **Section/topic** | **#** | **Checklist item** | **Reported on page #** |
| --- | --- | --- | --- |
| Risk of bias across studies | 15 | Specify any assessment of risk of bias that may affect the cumulative evidence (e.g., publication bias, selective reporting within studies). | Please refer to Screening and data extraction of the manuscript and Table S2. Quality review of articles included. We did not carry this out as it was not the intention of the review. |
| Additional analyses | 16 | Describe methods of additional analyses (e.g., sensitivity or subgroup analyses, meta-regression), if done, indicating which were pre-specified. | Screening and data extraction; ICF content analysis |
| **RESULTS** | | |  |
| Study selection | 17 | Give numbers of studies screened, assessed for eligibility, and included in the review, with reasons for exclusions at each stage, ideally with a flow diagram. | Results, Figure 1, Table S3 |
| Study characteristics | 18 | For each study, present characteristics for which data were extracted (e.g., study size, PICOS, follow-up period) and provide the citations. | Results, Figure 2, Table S2 and Table S3; study characteristics. |
| Risk of bias within studies | 19 | Present data on risk of bias of each study and, if available, any outcome level assessment (see item 12). | Table S2. Quality review of articles included |
| Results of individual studies | 20 | For all outcomes considered (benefits or harms), present, for each study: (a) simple summary data for each intervention group (b) effect estimates and confidence intervals, ideally with a forest plot. | We did not carry this out as it was not the intention of the review. |
| Synthesis of results | 21 | Present results of each meta-analysis done, including confidence intervals and measures of consistency. | We did not carry this out as it was not the intention of the review. |
| Risk of bias across studies | 22 | Present results of any assessment of risk of bias across studies (see Item 15). | Screening and data extraction. We did not carry this out as it was not the intention of the review. |
| Additional analysis | 23 | Give results of additional analyses, if done (e.g., sensitivity or subgroup analyses, meta-regression [see Item 16]). | N/A |
| **DISCUSSION** | | |  |
| Summary of evidence | 24 | Summarize the main findings including the strength of evidence for each main outcome; consider their relevance to key groups (e.g., healthcare providers, users, and policy makers). | Table S2. Quality review of articles included. Discussion section of the manuscript and study characteristics in results. |
| Limitations | 25 | Discuss limitations at study and outcome level (e.g., risk of bias), and at review-level (e.g., incomplete retrieval of identified research, reporting bias). | Table S2. Quality review of articles included. Discussion section of the manuscript and study characteristics in results. |
| Conclusions | 26 | Provide a general interpretation of the results in the context of other evidence, and implications for future research. | Discussion and conclusion sections of the manuscript |
| **FUNDING** | | |  |
| Funding | 27 | Describe sources of funding for the systematic review and other support (e.g., supply of data); role of funders for the systematic review. | No current funding sources for this study. |
